# Mechanistic Insights into the Capsule-Targeting Depolymerase from a Klebsiella pneumoniae Bacteriophage

**DOI:** 10.1128/spectrum.01023-21

**Published:** 2021-08-25

**Authors:** Rhys A. Dunstan, Rebecca S. Bamert, Matthew J. Belousoff, Francesca L. Short, Christopher K. Barlow, Derek J. Pickard, Jonathan J. Wilksch, Ralf B. Schittenhelm, Richard A. Strugnell, Gordon Dougan, Trevor Lithgow

**Affiliations:** a Infection and Immunity Program, Biomedicine Discovery Institute and Department of Microbiology, Monash University, Clayton, Australia; b Centre to Impact AMR, Monash University, Clayton, Australia; c Drug Discovery Biology Theme, Monash Institute of Pharmaceutical Sciences, Monash University, Parkville, Victoria, Australia; d Wellcome Sanger Institute, Hinxton, Cambridgeshire, United Kingdom; e Department of Medicine, University of Cambridge, Cambridge, United Kingdom; f Monash Proteomics & Metabolomics Facility, Biomedicine Discovery Institute and Department of Biochemistry and Molecular Biology, Monash University, Clayton, Australia; g Department of Microbiology and Immunology, The Peter Doherty Institute, The University of Melbourne, Parkville, Australia; Emory University School of Medicine

**Keywords:** *Klebsiella*, bacteriophages, capsular polysaccharide, cryo-EM, depolymerase

## Abstract

The production of capsular polysaccharides by Klebsiella pneumoniae protects the bacterial cell from harmful environmental factors such as antimicrobial compounds and infection by bacteriophages (phages). To bypass this protective barrier, some phages encode polysaccharide-degrading enzymes referred to as depolymerases to provide access to cell surface receptors. Here, we characterized the phage RAD2, which infects K. pneumoniae strains that produce the widespread, hypervirulence-associated K2-type capsular polysaccharide. Using transposon-directed insertion sequencing, we have shown that the production of capsule is an absolute requirement for efficient RAD2 infection by serving as a first-stage receptor. We have identified the depolymerase responsible for recognition and degradation of the capsule, determined that the depolymerase forms globular appendages on the phage virion tail tip, and present the cryo-electron microscopy structure of the RAD2 capsule depolymerase at 2.7-Å resolution. A putative active site for the enzyme was identified, comprising clustered negatively charged residues that could facilitate the hydrolysis of target polysaccharides. Enzymatic assays coupled with mass spectrometric analyses of digested oligosaccharide products provided further mechanistic insight into the hydrolase activity of the enzyme, which, when incubated with K. pneumoniae, removes the capsule and sensitizes the cells to serum-induced killing. Overall, these findings expand our understanding of how phages target the Klebsiella capsule for infection, providing a framework for the use of depolymerases as antivirulence agents against this medically important pathogen.

**IMPORTANCE** Klebsiella pneumoniae is a medically important pathogen that produces a thick protective capsule that is essential for pathogenicity. Phages are natural predators of bacteria, and many encode diverse “capsule depolymerases” which specifically degrade the capsule of their hosts, an exploitable trait for potential therapies. We have determined the first structure of a depolymerase that targets the clinically relevant K2 capsule and have identified its putative active site, providing hints to its mechanism of action. We also show that Klebsiella cells treated with a recombinant form of the depolymerase are stripped of capsule, inhibiting their ability to grow in the presence of serum, demonstrating the anti-infective potential of these robust and readily producible enzymes against encapsulated bacterial pathogens such as K. pneumoniae.

## INTRODUCTION

Klebsiella pneumoniae is a Gram-negative opportunistic pathogen found ubiquitously in the environment and as a commensal of humans and animals (1–3). Historically, K. pneumoniae infections tended to occur in immunocompromised people, including neonates and the elderly, leading to a range of diseases, including pneumonia, urinary tract infections, and septicemia (3, 4). However, with the increased prevalence of hypervirulent lineages, K. pneumoniae infections have now become a broader health care concern, even in immunocompetent people, with no effective treatment regimens for some panresistant isolates (5–7). As such, the World Health Organization placed K. pneumoniae in the group of critical priority pathogens requiring urgent new control strategies (8).

Bacteriophages (otherwise known as phages) are natural predators of bacteria, and phage therapy has long been considered a promising complementary strategy in combating antimicrobial-resistant (AMR) infections. To infect their bacterial hosts, phages must first bind a specific receptor on the bacterial cell surface (9). This targeting of a host bacterium is mediated by receptor-binding proteins (RBPs), such as tailspike proteins or tail fiber proteins, which recognize host cell surface structures to initiate infection. Phage can productively bind to the bacterial cell surface using receptor structures that include outer membrane proteins, such as beta-barrel porins, flagella, or pili. Alternatively, phage can bind carbohydrate surface structures: the lipopolysaccharide (LPS) in the outer leaflet of the outer membrane or the capsular polysaccharide (CPS) that is secreted out to form the outermost layer of the bacterial cell envelope (9). It is the specificity of the receptor-RBP interaction that limits the host range of an individual phage to a particular bacterial strain or species. In the less common cases of phages capable of infecting several hosts, multiple RBPs can be utilized, forming complex tail assemblies (10–12). In the case of Klebsiella and other encapsulated bacteria, the CPS is often the first surface structure a phage encounters and has often been identified as a determinant of phage specificity.

The thick CPS layer of K. pneumoniae is essential for virulence and forms a protective barrier surrounding the bacterial cell against harmful environmental factors such as antimicrobial compounds, phagocytosis by host immune cells, and infection by phages (3). K. pneumoniae produces at least 79 chemically diverse capsule serotypes, termed K antigens, which differ in the composition of sugars and the nature of glycosidic linkages present within the repeating unit of the polysaccharide. The enzymes responsible for this CPS diversity are encoded in a CPS locus of Klebsiella and have been characterized, and based on sequence analysis, 134 genotypes (KL types) have been described (13, 14). In most hypervirulent strains, the levels of capsule production are increased and this hypermucoviscous phenotype has most often been observed in capsule-type K1- or capsule-type K2-producing K. pneumoniae strains (15). To breach this protective barrier, phages enzymatically degrade the polysaccharide with “depolymerase” proteins (16), which act on O-glycosidic bonds and function either as hydrolases or lyases (17).

Based largely on homology modeling (16–18), but supported by recent structural analyses (11, 12), depolymerase RBPs are modular, comprising three functional domains: an N-terminal domain that tethers it to the phage baseplate or to other elements of the tail, a central β-helical domain that contains the enzymatic activity of the protein, and a C-terminal domain which may either act as an autochaperone or, in some cases, form a noncatalytic carbohydrate binding module. Within this overall framework, homology-based features of depolymerase RBPs are somewhat varied, with seven different structural scaffolds described (12, 18, 19). However, there are often limitations with sequence-informed predictions, especially given the overall lack of sequence homology in virion components (20).

The application of these phage-derived depolymerases as potential antivirulence agents has been widely investigated as a means to sensitize bacteria to antimicrobials and the immune system. Given the protective nature of the capsule, depolymerase-treated Klebsiella cells exhibit reduced survival in serum and demonstrate reduced disease outcomes in both mouse and Galleria mellonella larval models of infection (21–24). Some of the enzymes are also capable of degrading polysaccharides in a biofilm matrix (25, 26), which would allow the efficient penetration of antibiotics or cleaning agents to decontaminate surfaces such as medical devices and indwelling catheters.

In this study, we discovered and characterized the Klebsiella phage RAD2, which targets strains of K. pneumoniae that produce the clinically relevant K2 capsule. A transposon-directed insertion sequencing (TraDIS) functional genomics screen revealed that a select set of genes involved in the biosynthesis of CPS and the LPS core was required for RAD2 infection. Using cryo-electron microscopy (cryo-EM), we report the structure of the RBP depolymerase, identifying key features for its tethering to the virion and for its enzymatic activity. *In vitro* assays coupled with mass spectrometry approaches provided further mechanistic insights into the enzymatic activity of the depolymerase. Finally, we show that the recombinant form of this protein can efficiently strip K2 capsule from whole Klebsiella cells, which, in turn, show reduced growth in the presence of serum, providing a further framework for the potential use of depolymerases as antivirulence agents against K. pneumoniae.

## RESULTS

### RAD2 genome characteristics and virion architecture.

**FIG 1:**
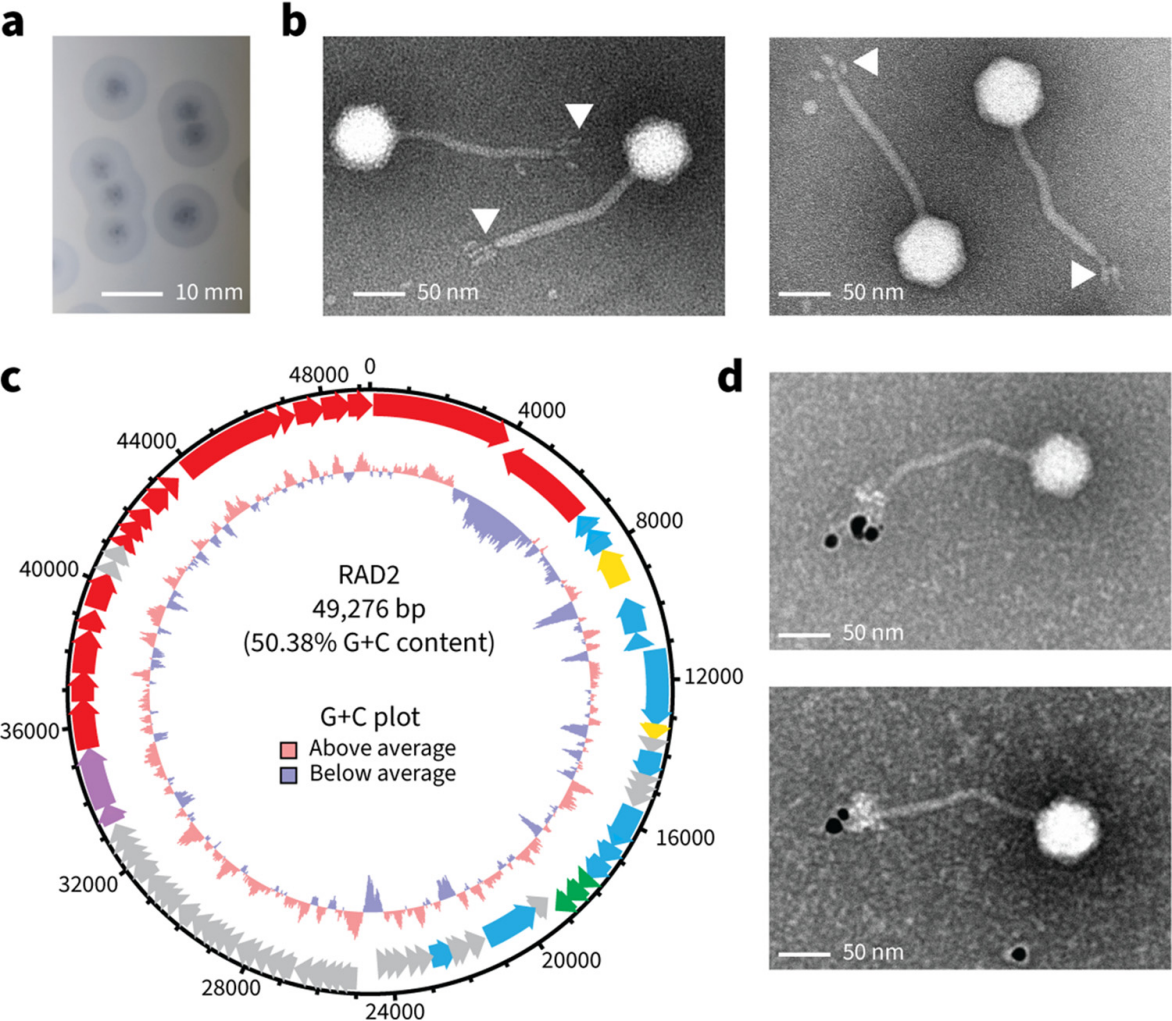
Isolation and characterization of Klebsiella phage RAD2. (a) Plaque morphology of RAD2 after infection of K. pneumoniae B5055. Scale bar, 10 mm. (b) Samples of phage RAD2 were purified using cesium chloride gradients and subjected to TEM analysis. White arrows highlight the globular tail appendages. Scale bar, 50 nm. (c) Circular representation of the RAD2 genome with putative functional assignment of the predicted open reading frames based on sequence analysis using BLASTp, HMMER, and HHpred color-coded by predicted function (pink, terminase; blue, DNA modification; yellow, nuclease; green, host cell lysis; red, structural or virion assembly; gray, unknown). (d) Representative images of cesium chloride-purified samples of RAD2 analyzed by immunogold labeling with antibodies raised against DpK2. Scale bar, 50 nm.

Klebsiella phage RAD2 was isolated from environmental water samples taken near Addenbrooke’s Hospital in Cambridge, United Kingdom, using K. pneumoniae B5055 as the host. RAD2 showed lytic activity and produced rough, clear plaques of ∼5 mm in diameter surrounded by a translucent halo (Fig. 1a). Transmission electron microscopy (TEM) of purified phage samples (Fig. 1b) revealed that the RAD2 virion is characteristic of siphoviruses, with an icosahedral capsid (∼65 nm in diameter) and a flexible noncontractile tail (∼160 nm in length). The tail displayed multiple appendages with dimensions of approximately 17 nm by 8 nm (Fig. 1b, white arrows).

Genome sequencing revealed that the RAD2 genome is 49,276 bp in length with a G+C content of 50.38% (GenBank accession no. MW655991). The genome is predicted to consist of 76 open reading frames (Fig. 1c; see Table S1 at https://figshare.com/s/ac477372e1a9d9f992b3), no tRNA genes (using tRNAscan-SE [27]), and to be terminally redundant and circularly permuted (using Phage Term [28]). Comparative analysis of phage whole-genome sequences showed that RAD2 belongs to the *Webervirus* genus, being most closely related to the Klebsiella phage GH-K3 (29) (see Fig. S1 at the URL mentioned above). The STEP^3^ (20) predictor identified 25 protein components that would be present in the virion, including a putative depolymerase DpK2 (*gp02*) (see Table S1 at the URL mentioned above). *gp02* encodes the 98-kDa DpK2 protein, which has domain features similar to those found in proteins annotated as tailspike or tail-fiber depolymerase proteins (see Tables S2 and S3, respectively, at the URL mentioned above). To determine the location of DpK2 on the RAD2 virion, polyclonal antibodies were raised against DpK2 and immunogold-labeling TEM was performed. Specific labeling of DpK2 was observed as the distal tail appendages of the RAD2 virion (Fig. 1d).

### Surface capsular polysaccharides serve as the major receptor for RAD2 targeting of K. pneumoniae.

An assessment of host range showed that RAD2 was only able to kill K. pneumoniae strains that produced a K2 capsule (see Table S4 at https://figshare.com/s/ac477372e1a9d9f992b3 for a complete list of tested strains). To determine the basis for this exclusive targeting of the K2-type CPS and to identify potential RAD2 receptors, we challenged a saturated transposon mutant library of K. pneumoniae ATCC 43816 (30) with bacteriophage RAD2 for 5 h and identified the surviving mutants by TraDIS. Fifteen genes were identified to be required for RAD2 infection (summarized in Table S5 at the URL mentioned above).

**FIG 2:**
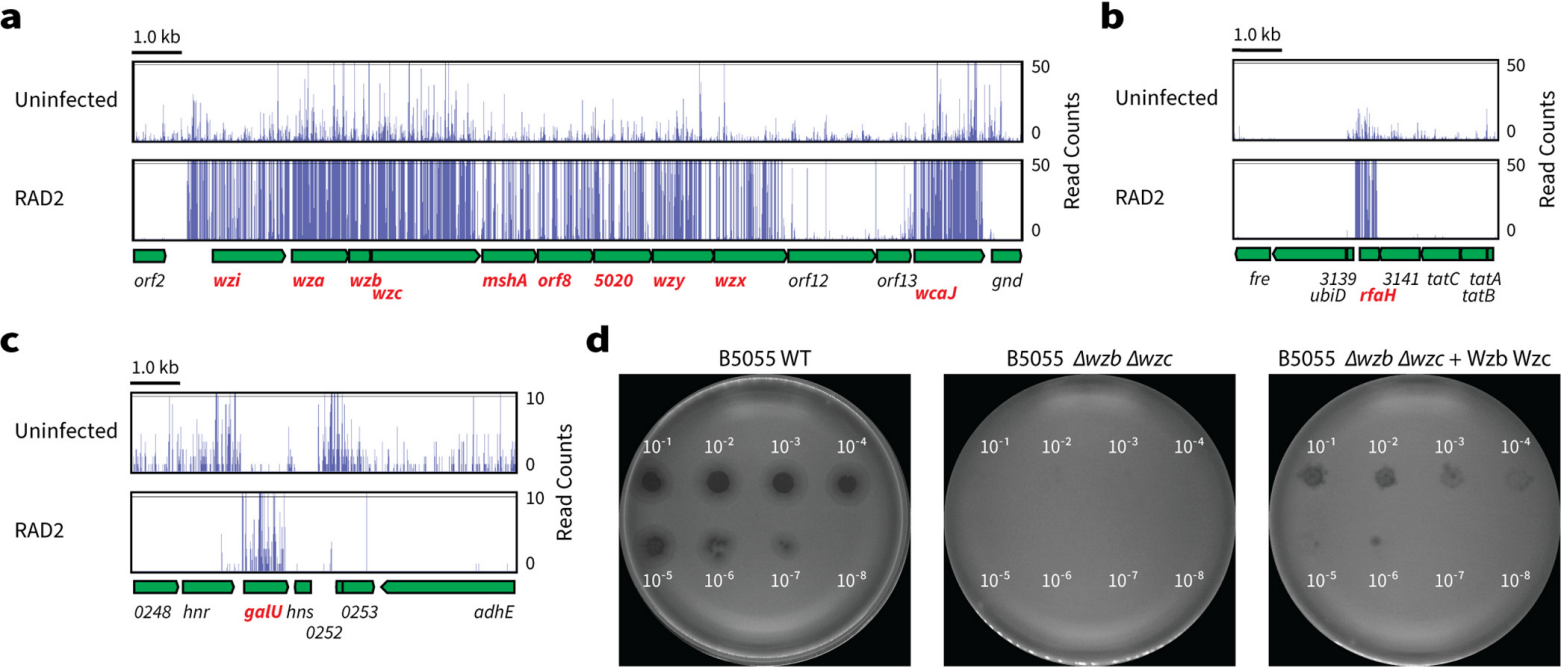
The K2 capsule is required for Klebsiella phage RAD2 infection. Summary of the TraDIS screen results. Transposon insertion sites mapped onto genes involved in CPS biosynthesis, export, and regulation. (a to c) An enrichment of insertions was observed in many genes (highlighted in red) of the CPS locus (a), the transcriptional antiterminator *rfaH* (b), and the UTP-glucose-1-phosphate uridylyltransferase *galU* (c) after infection by RAD2 compared to the uninfected input library. Scale bar, 1.0 kb. (d) Spot assays of diluted RAD2 preparations onto top agar layers containing K. pneumoniae B5055 wild type, B5055 Δ*wzb* Δ*wzc*, or the complemented B5055 Δ*wzb* Δ*wzc* mutant expressing plasmid-encoded Wzb and Wzc from an anhydrotetracycline-inducible promoter. The neat RAD2 titer used to prepare the serially diluted samples was ∼10^8^ PFU/ml.

The first set of genes necessary for RAD2 infection encode proteins involved in the biosynthesis of capsular polysaccharides. These included genes of the CPS locus (*wzi*, *wza*, *wzb*, *wzc*, *mshA*, *orf8*, VK055_5020, *wzy*, *wzx*, *orf12*, *orf13*, and *wcaJ*) (31) (Fig. 2a), the transcriptional antiterminator *rfaH* that activates CPS biosynthesis (32, 33) (Fig. 2b), and the UTP-glucose-1-phosphate uridylyltransferase gene *galU*, which contributes to the production of UDP-glucose and UDP-galactose, two of the major precursors of CPS biosynthesis (34, 35) (Fig. 2c). Note that while many genes are required for full capsule production or hypermucoidy in K. pneumoniae ATCC 43816 and its derivative K. pneumoniae KPPR1 (30, 36), only those genes where mutation is predicted to result in a complete loss of capsule were required for RAD2 infection. To validate the role of the capsule for RAD2 infection, an acapsular mutant of B5055 which lacks *wzb* and *wzc* was used as an infection host (37). Serially diluted RAD2 was spotted onto top agar containing either B5055, B5055 Δ*wzb* Δ*wzc*, or the complemented B5055 Δ*wzb* Δ*wzc* mutant expressing plasmid-encoded Wzb and Wzc. Visible RAD2 plaques were observed from samples diluted to 10^−7^ using the wild-type host but not the isogenic acapsular mutant (Fig. 2d). RAD2 sensitivity was partially restored in the complemented mutant, with smaller visible plaques forming from samples diluted to 10^−6^ (Fig. 2d). This suggests that the presence of capsule is essential for RAD2 infection and, taken together with the host range analysis of RAD2 (see Table S4 at https://figshare.com/s/ac477372e1a9d9f992b3), means that a K2-type capsule is both necessary and sufficient for RAD2 to select its bacterial prey.

The products of *rfaH* and *galU* are also relevant to the biosynthesis of LPS, and the second set of genes necessary for RAD2 infection encode additional proteins involved in the biosynthesis of LPS (see Fig. S2a at https://figshare.com/s/ac477372e1a9d9f992b3). These included the *N*-acetylglucosaminyl transferase gene *wabH* and the LPS-GlcNAc deacetylase gene *wabN*, both of which are required for the incorporation of α-GlcN into the LPS outer core (38, 39), and the LPS heptosyltransferase III gene *waaQ*, which attaches α-Hep III to α-Hep II within the LPS inner core (40). Although the LPS outer core structure varies between the TraDIS background strain ATCC 43816 (type 1) (41, 42) and the RAD2 isolation host B5055 (type 2) (40) (see Fig. S2b at the URL mentioned above), the glycosidic linkages in the substrates processed by WabH, WabN, and WaaQ are conserved. These findings are consistent with a model wherein LPS may serve as a secondary receptor targeted by RAD2.

### Depolymerase architecture and high-resolution structure of the tail appendages in phage RAD2.

**FIG 3:**
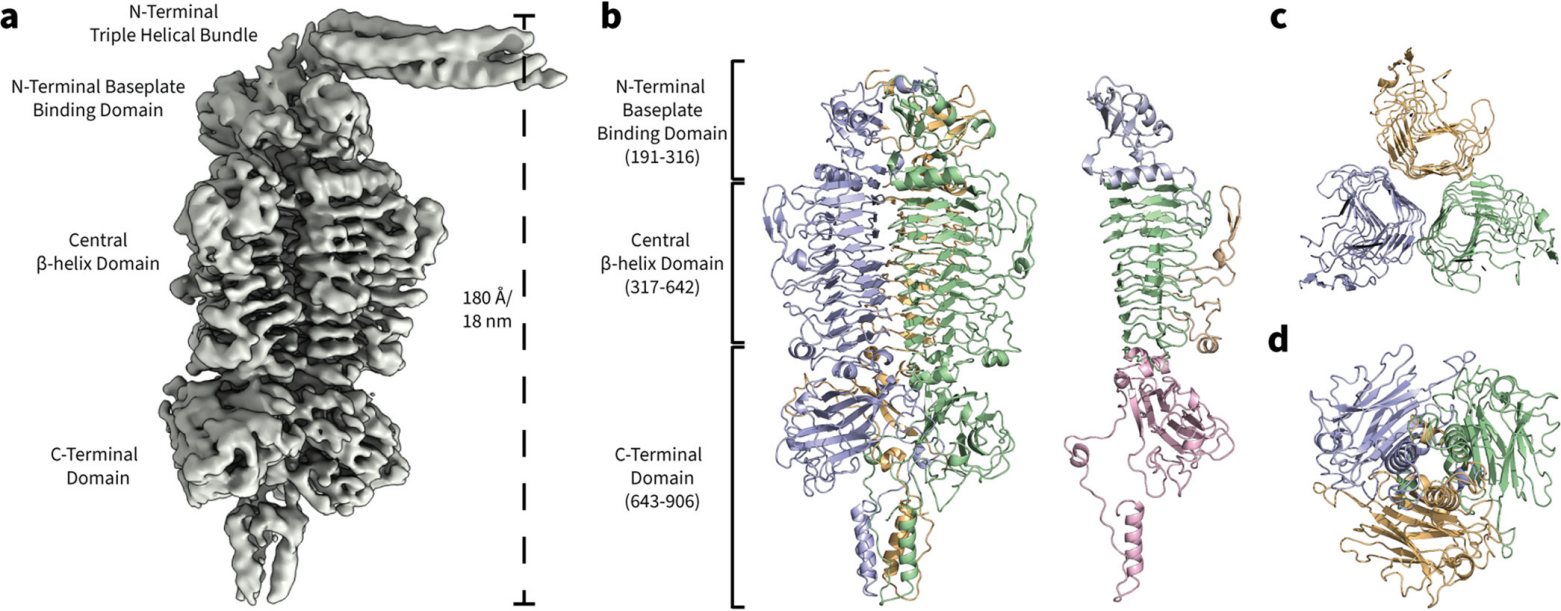
Structure of the tailspike protein DpK2 from Klebsiella phage RAD2. (a) Single-particle cryo-EM electron density of DpK2 refined using C1 symmetry. (b) High-resolution structure of the DpK2 trimer. The individual protein monomers are colored in green, blue, and yellow. Structural domains are highlighted on the Dpk2 monomer: N-terminal baseplate binding domain (residues 191 to 316, blue), the central β-helix domain (residues 317 to 642, green) with extended outward facing loop structures (yellow), and a C-terminal domain (residues 643 to 906, pink). (c) Cutaway top-down view of the three adjacent β-helices of the DpK2 trimer. Each helix is formed by an individual monomer assembled along the length of the protein. (d) Bottom-up view of the DpK2 trimer showing the C-terminal domain, which is formed predominantly by three individual β-sandwich folds and a short α-helical bundle forming a spike-like structure.

Given that the K2 capsule is essential for RAD2 infection, we sought to gain further insights into how RAD2 targets the capsule of its host. The putative depolymerase DpK2 protein was produced in recombinant form and purified via size exclusion chromatography (SEC), with a view to structural analysis. DpK2 was purified as a high-molecular-weight species of ∼300 kDa that, when analyzed by reducing sodium dodecyl sulfate-polyacrylamide gel electrophoresis (SDS-PAGE), migrated as a prominent band at ∼95 kDa (see Fig. S3a and b at https://figshare.com/s/ac477372e1a9d9f992b3), consistent with the native form of the protein being a trimer (Fig. S3c). Initial reconstructions using C1 symmetry revealed an elongated molecule of approximately 180 Å (18 nm) in length (Fig. 3a), consistent with the dimensions of the tail appendages measured in the initial TEM micrographs of the RAD2 virion (Fig. 1d). Three discernible domains of DpK2 could be observed: an N-terminal domain containing an extended triple helical bundle, a central beta-helical domain, and a distinct C-terminal domain (Fig. 3a).

C3 symmetry was used to further refine the structure of DpK2 to 2.7 Å resolution. Residues 191 to 906 of DpK2 could be modeled within the electron density. However, due to the applied C3 symmetry, high-resolution structural information of the N-terminal triple helical bundle was lost. The central domain (residues 317 to 642) is formed by three parallel right-handed β-helices consisting of 10 rungs (Fig. 3b), with each helix of the trimer being formed by an individual monomer (Fig. 3b and c). Long, extended loop structures were observed between several turns on the exposed faces of each β-helix of DpK2, which have been shown to form part of the oligosaccharide binding cleft of the RBP in phage P22 (43). The C-terminal domain (residues 643 to 906) is predominantly formed by three four-stranded β-sandwich folds, characteristic of oligosaccharide-binding domains (see Fig. S4 and Table S7 at https://figshare.com/s/ac477372e1a9d9f992b3), followed by a far C-terminal α-helical bundle resembling a spike-like structure (Fig. 3b and d).

DALI searches (44, 45) using the DpK2 structure identified similarities to several sugar-binding proteins such as the Azotobacter vinelandii mannuronan C-5 epimerase AlgE4 and the tailspike hydrolase proteins TSP2 and TSP4 from Escherichia coli O157:H7 phage CBA120 (see Table S6 and Fig. S4 at https://figshare.com/s/ac477372e1a9d9f992b3). These structure-based relationships were limited to the N-terminal and central β-helix domains of DpK2. Additional homology searches of the C-terminal domain (residues 643 to 906) of DpK2 showed similarities to domains of sugar binding enzymes such as the mouse peptide-N-glycanase PNGase (see Table S7 at the URL mentioned above); however, these proteins lacked the far C-terminal α-helical bundle which may be a specific feature of DpK2 (see Fig. S4 at the URL mentioned above). While the function of the DpK2 C-terminal domain is unknown, the structural similarities of this β-sandwich fold found within other polyoligosaccharide binding proteins would be consistent with this domain facilitating the initial binding of the enzyme to its target capsular polysaccharides.

### Enzymatic activity of DpK2.

**FIG 4:**
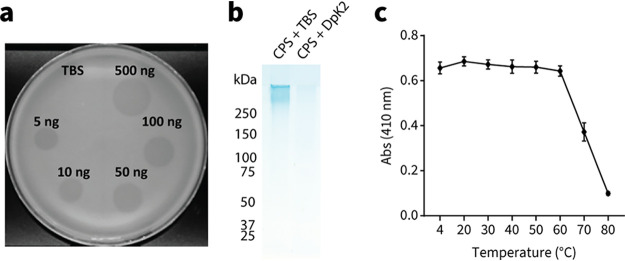
Recombinant DpK2 is an active enzyme. (a) Halo spot assays measuring the activity of DpK2 using double overlay plates. The top agar was preinoculated with K. pneumoniae B5055 before spotting it with increasing amounts of purified DpK2 (5 to 500 ng) or TBS. (b) Extracts of purified K2 capsule treated with either 1 μg of DpK2 or TBS analyzed by 3-to-14% SDS-PAGE and stained with Alcian blue. (c) Thermal stability of DpK2 measured with the colorimetric compound pHBAH. DpK2 (500 μg) was preincubated at the indicated temperatures for 30 min prior to the addition of purified K2 capsule. Absorbance readings at 420 nm were used to measure the generation of reducing sugars produced by the cleavage of capsular polysaccharide. Error bars represent standard deviations of the results of three biological replicates.

To measure enzymatic activity, purified DpK2 was titrated onto double overlay agar preinoculated with K. pneumoniae B5055. Translucent zones of clearance were observed within spots containing as little as 5 ng of DpK2 (Fig. 4a). Consistent with the host range of RAD2, zones of clearance produced by DpK2 were observed only for K2-producing strains (see Table S4 at https://figshare.com/s/ac477372e1a9d9f992b3), suggesting that the depolymerase may determine the host range of RAD2. To directly demonstrate K2 capsule degradation by DpK2, capsular polysaccharides were isolated from whole B5055 cells using phenol-water extraction (46) and subsequently incubated with either 1 μg of DpK2 or Tris-buffered saline (TBS). Samples were then analyzed by SDS-PAGE and stained with the cationic dye Alcian blue to visualize the negatively charged capsule (47). A high-molecular-weight species corresponding to the capsule was observed in samples incubated with TBS, which was absent in DpK2-treated samples (Fig. 4b). An assessment of the thermal stability of DpK2 was also undertaken using the colorimetric substrate pHBAH (*p*-hydroxybenzoic acid hydrazide) (48). Incubation of DpK2 for 30 min at temperatures greater than 70°C was required before the depolymerase would be rendered nonfunctional (Fig. 4c), and this high thermal stability is a common feature of phage tailspike proteins attributed to their conserved trimeric β-helical domain architecture (22, 49, 50).

**FIG 5:**
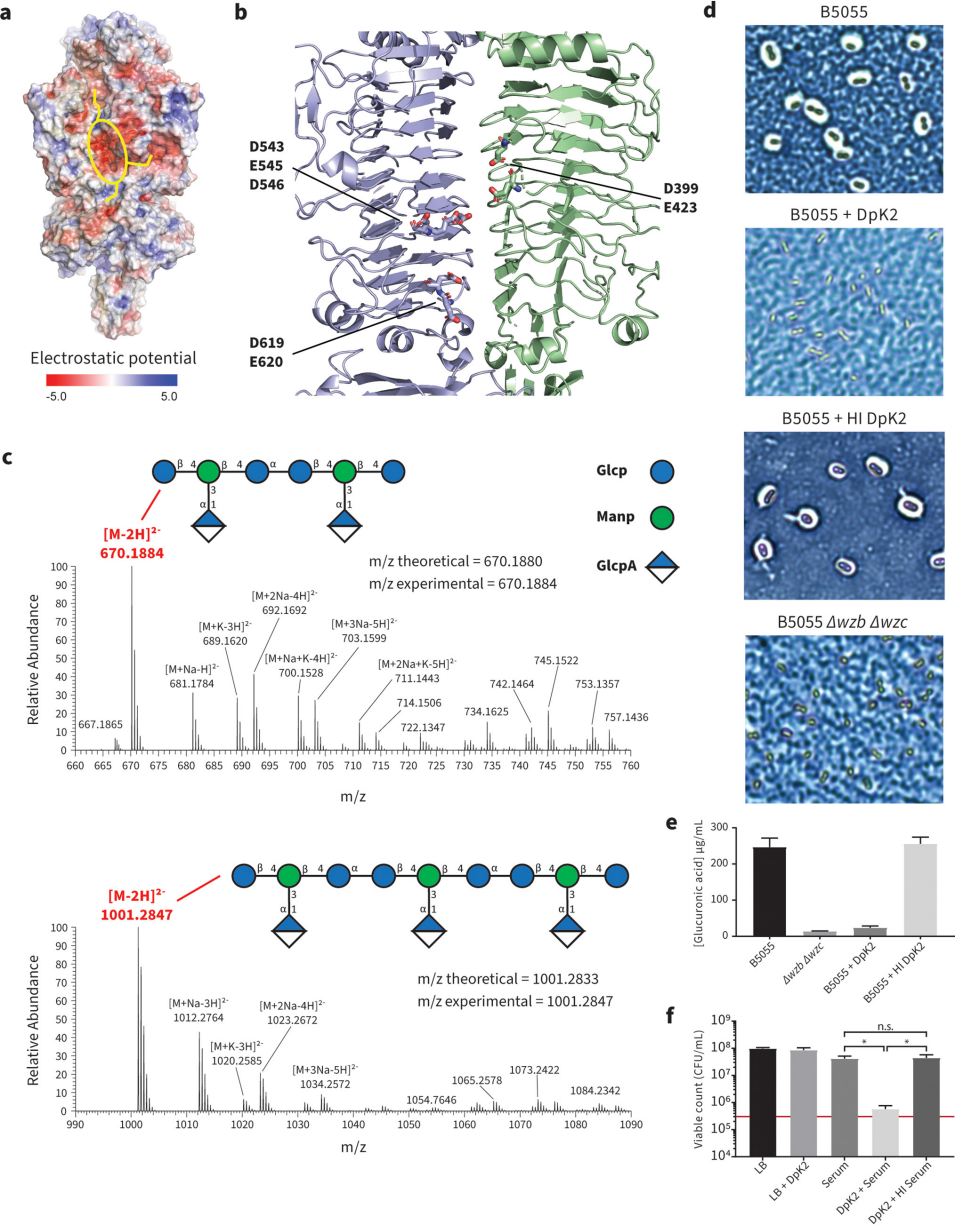
Mechanistic insight into the enzymatic activity of DpK2. (a) The electrostatic potential (negative, red; positive, blue) mapped onto the surface of the DpK2 trimer, highlighting a strong negatively charged cavity (yellow oval) connected to adjacent grooves (yellow lines). (b) The negatively charged cavity is formed within an interchain groove between neighboring DpK2 monomers. (c) Negative-mode mass spectra of DpK2-treated capsule analyzed by LC-MS. The prominent masses are highlighted in red. The multiple masses observed within these spectra can be attributed to the formation of ion adducts that differ in the nature of ionizing species. The cartoon structure of the K2 repeating units is depicted with the theoretical and experimental *m/z* ratios highlighted for both cleavage products. Glc, glucose; Man, mannose; GlcA, glucuronic acid; *p*, sugars in pyranose form. (d) Representative images of enzyme-treated K. pneumoniae cells visualized by Maneval’s staining and light microscopy. (e) Quantification of cell-associated uronic acid (μg/ml). Capsular polysaccharides were isolated from whole cells using phenol-water extraction after treatment with active or heat-inactivated DpK2. Samples were hydrolyzed in sulfuric acid prior to the addition of 3-hydroxyldiphenol and subsequent absorbance reading at 520 nm. The uronic acid concentration was measured using a defined glucuronic acid standard curve. Error bars represent standard deviations of the results of three biological duplicates. (f) Quantitation of K. pneumoniae B5055 growth in the presence of DpK2 and serum as measured by the number of recovered CFU/ml. Cells were treated with either DpK2, serum, DpK2 plus serum, or DpK2 plus heat-inactivated (HI) serum for 3 h. The red bar represents the number of CFU/ml of the starting inoculum prior to the addition of serum. Overall statistical significance was determined by one-way analysis of variance (ANOVA) (*P* value < 0.0001 for all conditions). Further statistical analyses were performed using Turkey’s multiple-comparison test for all serum treatments (*, *P* < 0.0001; n.s., not significant). Error bars represent standard deviations of the results of three biological duplicates.

We next sought to determine how DpK2 degrades the K2 capsule. Semantically, “depolymerase” suggests a processive degradation of a polymer, whereas phage RBP depolymerases function as either hydrolases or lyases (16, 17). In the case of hydrolases, a negatively charged cavity formed by aspartic or glutamic acid pairs is required for the hydrolysis of target substrates (51). Analysis of the surface properties of DpK2 revealed a prominent negatively charged cavity (Fig. 5a, yellow oval) located within the central β-helical domain at the interface between neighboring monomers. Adjacent grooves (Fig. 5a, yellow lines) were observed emanating from the cavity, which may facilitate binding to or funneling of substrates toward the active site. This cavity consists of a negatively charged triad (D543, E545, and D546) and two dyads (D399-E423 and D619-E620) within close proximity, suggesting that DpK2 may function as a hydrolase (Fig. 5b). To test this hypothesis, we applied mass spectrometry to analyze the products of the enzymatic reaction.

The repeat unit of the K2 capsule has a main chain polysaccharide with the sequence -3)-β-d-Glcp-(1-4)-β-d-Manp-(1-4)-α-d-Glcp-(1-, and a single glucuronic acid (α-d-GlcpA) side chain residue bound to the mannose residue through a (3-1) linkage (52) (see schematic in Fig. 5c). To characterize the glycosidic cleavage of the K2 capsule by DpK2, purified enzyme was incubated with purified capsule and the resulting mixture was analyzed by high-resolution liquid chromatography-mass spectrometry (LC-MS). This analysis revealed two sets of LC-MS features which were absent in the corresponding heat-inactivated enzyme control. Specifically, these features correspond to either two or three repeat units of the K2 capsule, each of which is detected predominately as the doubly deprotonated anion, with several additional peaks corresponding to combinations of sodium and potassium adducts. These oligosaccharide products were in good agreement with the theoretical masses of hydrolytically processed K2 oligosaccharides. However, due to the isomeric nature of the sugars within the K2 backbone, the precise cleavage site cannot be identified.

To address how effective the hydrolase activity of DpK2 is against whole Klebsiella cells, we used Maneval’s staining (53). The capsule of B5055 is extensive, radiating ∼375 nm out from the cell surface as judged by atomic force microscopy (54). Maneval’s staining showed extensive removal of capsule (Fig. 5d), which was consistent with independent assays directly measuring the amount of cell-associated glucuronic acid after enzymatic treatment (Fig. 5e). In both assays, treatment of B5055 with DpK2 made it comparable to the acapsular mutant strain and heat inactivation of the enzyme inhibited this loss of capsule. CPS enables B5055 to avoid complement-mediated killing, with *wzb wzc* mutants showing loss of viability in the presence of human serum (see Fig. S5 at https://figshare.com/s/ac477372e1a9d9f992b3). In assays of K. pneumoniae B5055, neither DpK2 alone nor serum alone significantly decreased cell viability as determined by colony counts, but when incubated together, the reduction in capsule caused by DpK2 inhibited viability of B5055 in the presence of serum (Fig. 5f).

## DISCUSSION

Surface polysaccharides of K. pneumoniae, both LPS and CPS, are essential virulence factors that protect the bacterial cell from harmful environmental stimuli and show extensive complexity in their sugar compositions and linkages (3, 13, 14). As natural predators of bacteria, phages have evolved mechanisms to specifically recognize and enzymatically degrade these protective barriers, allowing for subsequent infection of their host (9). While the archetypal siphoviruses such as Lambda, T1, T5, and Chi use “L-shaped” or fibrous appendages on their tails to bind cell surface features (55–59), phage RAD2 has globular appendages comprised of the depolymerase RBP DpK2. Here, we characterized the structure and enzymatic activity of DpK2, showing it to be a capsule-degrading hydrolase specific for K2-type polysaccharides.

Our data suggest that the K2-type capsule is both necessary and sufficient for infection by phage RAD2. CPS was identified as an essential receptor through loss-of-function studies using TraDIS, with genes of the CPS locus, the transcriptional antiterminater *rfaH*, and the UTP-glucose-1-phosphate uridylyltransferase *galU* found to be necessary for RAD2 infection. This was confirmed by the resistance of an independent isogenic acapsular mutant of K. pneumoniae B5055 to the phage. Additional hits were also observed to three genes, *wabH*, *wabN*, and *waaQ*, involved in the biosynthesis of the LPS core. We cannot be certain that the LPS core is recognized by a second, as yet unidentified, phage RBP as a secondary receptor. An alternative model is that LPS is a secondary receptor only in the sense that it maintains the structure of the primary receptor, CPS. Transposon mutants lacking a functional copy of *wabH*, *wabN*, or *waaQ* have a diminished level of capsule retained on their cell surface (30), and it has been demonstrated that aspects of the LPS core are directly involved in capsule retention in K. pneumoniae 52145 (40, 60).

To investigate the mechanism by which the RBP capsule depolymerase functions, DpK2 was purified as an enzymatically active trimer and its structure solved at a resolution of 2.7 Å. DpK2 consists largely of a central characteristic β-helical domain architecture with a putative active site being formed within a highly negatively charged intersubunit groove. Architecturally, DpK2 shows the highest structural homology to the tail fiber LPS hydrolase TSP2 from the E. coli phage CBA120. However, structural differences were observed in the C-terminal domain of DpK2, which consists of a β-sandwich fold followed by a far C-terminal α-helical bundle. The role of this additional structural feature of DpK2 is unknown, but given the homology observed within the β-sandwich fold, it could be suggested that this domain forms a carbohydrate binding module commonly found in sugar binding proteins. Of the known Klebsiella phage targeting RBP depolymerases, only the K21-targeting depolymerase gp38 from the phage KP32 has been structurally characterized, highlighting the key catalytic residues required for CPS cleavage (12). Despite not sharing any significant sequence similarity, both proteins exhibit negatively charged patches within their intermolecular active sites suggestive of polysaccharide hydrolase activity. Given the chemical complexity of the Klebsiella capsule and the significant sequence diversity observed in RBP depolymerases, further detailed structural analyses of depolymerases are vital in understanding how phage-encoded enzymes target these protective surface polysaccharides.

Targeting bacterial surface polysaccharides by antibody therapies or by vaccines has shown some promise in combating infections by encapsulated bacterial pathogens such as K. pneumoniae (61). Given the essential role of the capsule for Klebsiella virulence, disrupting this protective barrier by other agents such as phages or phage-derived enzymes has gained interest as a complementary anti-infective strategy against these difficult-to-treat bacterial pathogens. Recombinantly produced DpK2 has potential antivirulence activity, capable of stripping the K2 capsule from whole Klebsiella B5055 cells. The clinically prevalent K2 capsule plays an essential role in complement resistance and in B5055 has been shown to be sufficient for survival in serum (62). More broadly, studies have also shown that depolymerase-treated K. pneumoniae cells are more easily cleared in mice and Galleria mellonella larval infection models (21, 22, 24, 63). Taken together, this study demonstrates the utility of undertaking a multidisciplinary approach to these often overlooked and underexploited phages, and their component parts, as we search for new tools to combat the rise of AMR in bacteria.

## MATERIALS AND METHODS

### Bacterial strains and phage isolation.

Host range analysis of phage RAD2 was performed and strain specificity of the protein DpK2 was determined using the strains listed in Table S4 at https://figshare.com/s/ac477372e1a9d9f992b3. The protein expression strain E. coli BL21 DE3* was used for production of DpK2 for structural analysis and antibody preparation.

Wastewater samples taken from waterworks associated with Addenbrooke’s Hospital, Cambridge, United Kingdom, were used for phage isolation, using K. pneumoniae B5055 as the host. Samples (50 ml) were mixed with 50 ml of 2-fold concentrated Luria-Bertani (LB) medium and 2 ml of a bacterial overnight culture and grown for a further 16 h at 37°C. Cellular debris was pelleted by centrifugation at 5,000 × *g* for 10 min, and the resulting supernatant was passed through a 0.45-μm filter. To monitor phage activity, 20 μl of the supernatant was then spotted onto LB agar plates containing a top layer of top agar (4 ml LB medium and 0.35% [wt/vol] agar) and 200 μl of bacterial culture and incubated overnight at 37°C. Subsequent liquid or spot test infections were performed as described previously (64). Infections of the Wzb- and Wzc-expressing cells were performed as described above with the following changes. Cells were subcultured 1:50 into 30 ml of fresh LB medium and grown at 37°C to an optical density (at 600 nm) of 0.3. The expression of Wzb and Wzc was induced with 30 ng/ml anhydrotetracycline, and cells were grown for a further 5 h before infection with RAD2 as described above.

### Generation of high-titer phage stocks.

Working RAD2 stocks were generated by performing liquid infections as described above. Four milliliters of SM buffer (100 mM NaCl, 8 mM MgSO_4_, 10 mM Tris, pH 7.5) was added to two 90-mm infection plates showing semiconfluent plaques and were incubated at 4°C for 1 h. The top agar layer was scraped off, and 50 μl of chloroform was subsequently added before vortexing rigorously for 1 min. The agar and cell debris were pelleted at 4,000 × *g* for 30 min at 4°C, and the resulting supernatant was passed through a 0.45-μm filter and stored at 4°C. To generate pure, high-titer RAD2 phage stocks, large-scale infections using 140-mm petri dishes were performed using 60 μl of phage preparation (10^−4^ dilution of the RAD2 working stock), 500 μl of an overnight culture of K. pneumoniae B5055, and 10 ml of top agar in two batches of 20 infections. RAD2 lysates were prepared from the pooled infection plates and purified by CsCl density gradients as previously described (55) prior to dialyzing them twice with 2 liters of SM buffer to remove residual CsCl from the sample.

### Phage gDNA extraction, sequencing, and annotation.

Phage genomic DNA (gDNA) was isolated from 1.8 ml of the RAD2 working stock (∼10^10^ PFU/ml). Eighteen microliters of DNase (1 mg/ml) and 8 μl of RNase A (12.5 mg/ml) were added and incubated at 37°C for 30 min. Eighteen microliters of proteinase K (10 mg/ml) and 46 μl of SDS (20% stock solution) were added to the lysate and subsequently incubated for a further 30 min at 37°C. Genomic DNA was isolated from the lysate by phenol-chloroform extraction using Phase Lock Gel tubes (QuantaBio catalog no. 2302820) as described previously (64). Whole-genome sequencing was performed as 2 × 250 bp paired-end reads using an Illumina MiSeq system by the NPG, DNA Pipelines Informatics Group, Wellcome Trust Sanger Institute. The RAD2 genome was assembled *de novo* using the Iterative Virus Assembler (65) and annotated by Prokka (66). The consensus sequence was then subsequently screened against the GenBank database using BLAST (67) (https://blast.ncbi.nlm.nih.gov/Blast.cgi).

### Sequence analysis.

Screening of virion-associated and nonvirion proteins made use of STEP^3^ (20), and protein sequence and domain analysis of each predicted RAD2 ORF was performed using BLAST and HMMer 3.3, respectively. Structural predictions were performed using HHpred (68) using the default settings. The circular genome representation and %G+C plots were generated using DNA plotter (69). Proteomic tree analysis and whole-genome alignments of RAD2 were performed using ViPTree (70) by use of the double-stranded DNA (dsDNA) nucleic acid type and Prokaryote host category database, which also included the genome of Klebsiella phage GH-K3. Refined trees were regenerated to analyze the phylogeny of “*Siphoviridae-*like” phages that infect *Gammaproteobacteria*.

### High-throughput genomics approach to identify essential host genes for RAD2 infection.

A high-density transposon mutant library of K. pneumoniae ATCC 43816 containing ∼250,000 unique insertion sites (30) was used to define mutants surviving treatment with RAD2. Three cultures were grown in LB medium, each inoculated with 10^9^ bacterial cells from the transposon library stock and 10^10^ RAD2 viral particles. Cultures were grown for a further 5 h, pelleted by centrifugation (6,000 × *g*, 10 min, at 4°C), and washed in 10 mM Tris. Genomic DNA was isolated from each cell pellet by phenol-chloroform extraction using 15-ml phase lock tubes (Qiagen). Two micrograms of each gDNA preparation was used to prepare transposon-specific sequencing libraries using primer FS108 for specific amplification of transposon junctions as described previously (30, 71). DNA libraries were sequenced using the Illumina MiSeq platform with primer FS107 as described previously (30).

TraDIS analysis was performed essentially as described previously (30, 71). Reads from transposon-gDNA junctions were mapped to the K. pneumoniae ATCC 43816 genome (GenBank accession no. CP009208) using the BioTraDIS pipeline with the parameters “-v smalt_r -1 -t TAAGAGACAG -mm 1” and assigned to genomic features, with reads mapping to the 3′ 10% of the gene ignored. Comparisons between phage-treated and control samples were performed using the “TraDIS_comparison_positive_selection.R” script (https://github.com/francesca-short/tradis_scripts), which is based on the comparison script from the BioTraDIS toolkit but in addition reports the insertion index ratio between condition and control samples. Filtering based on gene-wise transposon mutant diversity (insertion index ratio) was necessary because, for many of the genes with increased read counts post-phage challenge, these reads mapped to just a single insertion site. These cases were presumed to result from rare secondary mutations unrelated to the transposon insertion, as suggested previously (72). Genes required for phage infection were defined as those with a log2 fold change (FC) of  >1, a *q* value of <0.01, and an insertion index ratio of ≥1 between the phage-treated and input samples.

### Protein purification and antibody production.

The open reading frame (ORF) for DpK2 (gp02) was cloned with an N-terminal hexahistidine tag into the protein expression vector pPROEX htb (Thermo Fisher Scientific) using the primers RAD2_DpK2F – GCGCGCCATGGGCGCACTATACAGAGAAGGTAAAG and RAD2_DpK2R – CGCCGCTCGAGTTAGCTACTCATAAATCCATTTG. The underlined nucleotides represent the NcoI and XhoI restriction sites, respectively. The expression construct was transformed into E. coli BL21 DE3* (Novagen), cells were subsequently grown in 1 liter of Terrific Broth (12 g tryptone, 24 g yeast extract, 4 ml glycerol, 2.31 g KH_2_PO_4_, and 12.84 g K_2_HPO_4_ per liter) at 37°C until the cultures reached an optical density at 600 nm (OD_600_) of 0.8, and protein expression was induced with 0.3 mM IPTG (isopropyl-β-d-thiogalactopyranoside), with shaking overnight at 18°C. Cells were collected and lysed in lysis buffer (50 mM Tris [pH 8.0], 400 mM NaCl, 2 mM MgCl_2_, and 20 mM imidazole) using an Avestin Emulsiflex C3 cell press (3 passes). His-tagged proteins were first purified by application of a soluble lysate fraction to Ni-affinity chromatography, with lysis buffer used for binding to the 5-ml nickel HisTrap HP column (GE Healthcare) and, following washing, elution using a gradient of 50 mM Tris pH 8.0, 400 mM NaCl, and 1 M imidazole. Proteins were further purified by size exclusion chromatography using a HiLoad 16/600 Superdex 200-pg column (GE Healthcare) equilibrated in 25 mM Tris (pH 8.0) and 200 mM NaCl. Protein present in each peak was assessed by Coomassie blue-stained reducing SDS-PAGE and activity assays. DpK2 rabbit polyclonal antibody was generated to the purified recombinant protein at the Monash Animal Research Platform (MARP) in adherence to their ethics-approved protocols for generating antibodies in rabbits (this work is specifically covered by ERM project no. 14152).

### Transmission electron microscopy.

Purified phage preparations (4 μl) were added to freshly glow-discharged CF200-Cu carbon support film 200-mesh copper grids (ProSciTech) for 30 s. The sample was blotted from the grid using Whatman filter paper, and samples were subsequently stained with 4 μl of Nano-W methylamine tungstate (Nanoprobes) for 30 s and blotted again. Grids were imaged using a Tecnai Spirit G2 transmission electron microscope (Tecnai).

Immunogold labeling of DpK2 was performed as described previously, with the following changes (64). Freshly glow-discharged CF200-Cu carbon support film 200-mesh copper grids were incubated on a drop of CsCl_2_-purified RAD2 stock solution for 15 s. Excess liquid was removed using Whatman filter paper, and grids were immediately incubated on a drop of antibody solution (1:1,000 dilution of anti-DpK2 in 5% bovine serum albumin [BSA] in Tris-buffered saline [TBS]) for 20 min. Grids were rinsed briefly by transferring them onto two consecutive drops of TBS with 0.01% Tween, followed by a third drop of TBS only. Excess liquid was removed using Whatman filter paper, and the grids were then incubated on a drop containing 5-nm protein A-gold (Nanocs) for 20 min and subsequently washed as described above, finishing on a drop of distilled water. Excess liquid was blotted away, and grids were stained with Nano-W for 30 s, blotted, dried, and imaged using a 120 kV Tecnai Spirit G2 transmission electron microscope (Tecnai).

### Cryo-EM.

Purified DpK2 protein samples were diluted to 0.4 mg/ml in TBS and prepared for cryo-electron microscopy (cryo-EM) as described previously (73). Data were collected on a Glacios microscope (Thermo Fisher Scientific) operated at an accelerating voltage of 200 kV with a 70-μm C2 aperture at an indicated magnification of ×120,000 in nanoprobe energy-filtered TEM (EFTEM) mode, spot size 4. A Falcon 3 direct electron detector was used to acquire dose-fractionated images of the DpK2 complex. Movies were recorded as uncompressed gain-normalized .MRC files yielding a physical pixel size of 0.895 Å/pixel. An exposure time of 40 s amounting to a total dose of 43 e-/Å^2^ was fractionated into 38 subframes. Defocus was varied in the range between −0.4 to −1.5 μm. Beam-image shift was used to acquire data from 9 surrounding holes, after which the stage was moved to the next collection area using the EPU software package (Thermo Fisher Scientific).

### Data processing.

Movies were processed as described previously (73). Particles were initially picked from the micrographs using the Laplacian of Gaussian (LoG) automatic picker in RELION 3.0 (74–79). Extracted particles from the LoG-picked data set were then imported into CryoSPARC (version 2.9) (80) for reference-free two-dimensional (2D) classification and *ab initio* model generation. This *ab initio* model was then used for template-based picking and as an initial model in RELION. Particle extraction and reference-free 2D classification were carried out in RELION (version 3.0.7). A homogeneous subset of particles was then subjected to Bayesian particle polishing and contrast transfer function (CTF) refinement as implemented in RELION 3.1 (see Fig. S6 at https://figshare.com/s/ac477372e1a9d9f992b3 for the cryo-EM workflow), and each beam tilt group had its coma aberrations calculated separately in RELION-3.1. This homogeneous subset of polished particles was used for both a C3 symmetric and C1 asymmetric 3D refinement in RELION. The final resolutions estimated by using the gold standard forward scatter (FSC = 0.143) were 2.7 Å for the C3 refinement and 2.8 Å for the C1 refinement (see Fig. S7 at the URL mentioned above). Local resolution estimations were performed using RELION. The cryo-EM data collection and structure statistics are listed in Table S8 at the URL mentioned above.

### Atomic model refinement.

An initial atomic model was generated using the C3 symmetrized map using the Buccaneer software package (81). The model was then manually curated using the coot software package (82). Further model refinement was done using real-space refinement as implemented in Phenix (83, 84). The N-terminal domain near the C3 symmetry break was difficult to interpret, even in the asymmetrically refined maps, and the N-terminal triple-helix bundle and linker were not modeled.

### Capsule extraction and quantification.

Ten-milliliter cultures of K. pneumoniae B5055 were grown in LB medium to an OD_600_ of ∼0.5 before harvesting by centrifugation (6,000 × *g*, 10 min, at 4°C). Cells were subsequently washed with 1 ml Milli-Q H_2_O, pelleted (14,000 × *g*, 10 min, at 4°C), and resuspended in 500 μl Milli-Q H_2_O. Samples were incubated at 68°C for 2 min before the addition of 500 μl of phenol, mixed by inverting 20 times, and incubated at 68°C for 30 min. The samples were cooled on ice before the addition of 500 μl of chloroform, mixed by inverting, and centrifuged at 10,000 × *g* for 5 min. The aqueous phase (400 μl) containing the capsular polysaccharides (CPS) was precipitated with 40 μl 3 M sodium acetate and 1 ml cold 100% ethanol before inverting as described above and were then subsequently incubated at –20°C for 30 min. The samples were pelleted by centrifugation (16,000 × *g*, 10 min, 4°C), washed with 70% ethanol, air dried, and then resuspended with 500 μl Milli-Q H_2_O.

Purified CPS (100 μl) was added to 600 μl 12.5 M tetraborate dissolved in 18 M sulfuric acid, vortexed vigorously, and boiled for 5 min. The samples were cooled to room temperature before the addition of 10 μl 0.15% 3-hydroxyldiphenol in 0.5% sodium acetate and aliquoted to a 96-well plate, and the absorbance was measured at 520 nm. Standard curves were determined using 100 μl of glucuronic acid (0 to 200 μg/ml) as described above.

### SDS-PAGE and Alcian blue staining.

Ten micrograms of purified CPS was incubated with either 1 μg of purified DpK2 or an equivalent volume of TBS for 1 h at 37°C. Samples were analyzed by 3-to-14% gradient sodium dodecyl sulfate-polyacrylamide gel electrophoresis (SDS-PAGE) and subsequently stained with the cationic dye Alcian blue (47). Briefly, the gel was washed in fixing buffer (25% ethanol and 10% acetic acid in Milli-Q water) three times at 50°C (10 min each wash), before staining with 0.125% Alcian blue in fixing buffer (for 15 min at 50°C in the dark). The gel was destained with fixing buffer at room temperature and visualized.

### Depolymerase activity assays.

The activity of DpK2 was determined by measuring the production of reducing sugars upon CPS cleavage using the colorimetric compound *p*-hydroxybenzoic acid hydrazide (pHBAH) (48). A stock solution of 5% (wt/vol) pHBAH was prepared in 0.5% (vol/vol) HCl. A working solution of pHBAH was prepared fresh for each experiment by diluting the stock 1:4 (vol/vol) in 0.5 M NaOH. Prior to CPS digestion, aliquots of DpK2 were incubated at different temperatures (4 to 80°C) for 30 min. Fifty micrograms of purified CPS was treated with 500 ng of each DpK2 preparation and made up to a final volume of 25 μl with TBS, pH 8.0. Samples were subsequently incubated at 37°C for 15 min to allow digestion of the CPS and added to 75 μl of pHBAH working solution in a 96-well PCR plate. Samples were incubated at 95°C for 5 min in a thermocycler and allowed to cool to room temperature, and the absorbance was measured at 410 nm.

### LC-MS.

For mass spectrometry analyses of reaction products, purified enzyme (1 μg) was incubated with 100 μg of purified capsule at 37°C for 2 h. Ten microliters of the resulting mixture was diluted with the addition of 90 μl of water and passed through a preconditioned 30-kDa-molecular-weight-cutoff filter (Nanosep 30 kDa Omega; Pall Life Sciences) according to the manufacturer’s instructions. The filtrate was added to 400 μl acetonitrile, the resulting solution was centrifuged (22,000 × *g*, 4°C, 10 min), and the supernatant was analyzed by LC-MS. Samples in which the enzyme was either excluded or heat inactivated prior to incubation with the purified capsule were also prepared to act as negative controls.

LC-MS analysis utilized a zwitterionic hydrophilic interaction LC (HILIC) column (150 by 4.6 mm ZIC-pHILIC 5 μm, polymeric; Merck) protected by a guard column (20 by 2.1 mm ZIC-pHILIC 5 μm, polymeric; Merck) at 25°C with a gradient elution of 20 mM ammonium carbonate (A) and acetonitrile (B) (linear gradient time–%B as follows: 0 min–80%, 15 min–50%, 18 min–5%, 21 min–5%, 24 min–80%, 32 min–80%) at 300 μl/min on a Dionex RSLC3000 ultra-high-performance LC (UHPLC) (Thermo Fisher Scientific). Samples were kept at 6°C in the autosampler, and 10 μl was injected for analysis. Mass spectrometric analysis was performed at 35,000 resolution (accuracy calibrated to <1 ppm) on a Q-Exactive Orbitrap MS (Thermo Fisher Scientific) operating in rapid switching positive (4 kV)- and negative (−3.5 kV)-mode electrospray ionization (capillary temperature, 300°C; sheath gas flow rate 50; auxiliary gas flow rate 20; sweep gas flow rate 2; probe temperature, 120°C).

The LC-MS data were processed by applying the first three steps of the IDEOM v20 (85) workflow. Specifically, data corresponding to each polarity were extracted to separate files in .mzXML format using MSConvert (86). Untargeted extraction of chromatographic features (corresponding to a specific mass) and alignment were then performed using the XCMS algorithm (87) and mzMatch, respectively, implemented through R (R Core Team, 2013; http://www.R-project.org/). Features that were observed in the enzyme and capsule sample but not in the corresponding negative controls were then identified and manually assigned.

### Maneval’s staining of Klebsiella cells.

Cultures of K. pneumoniae wild-type strain B5055 or a nonmucoid mutant (Δ*wzb* Δ*wzc*) were grown in LB medium to an OD_600_ of ∼0.5. Fifty microliters of cells was aliquoted to microcentrifuge tubes before the addition of 100 μg/ml of active or heat-inactivated (incubated at 80°C for 30 min) DpK2 or an equivalent volume of TBS. Samples were then incubated at 37°C for 1 h. Three microliters of each culture was spotted onto a clean, grease-free microscope slide. Cell-associated capsule was observed by Maneval’s staining (53), and cells were visualized at ×100 magnification using the EVOS FL autoimaging system (Thermo Fisher Scientific).

### Serum sensitivity of depolymerase-treated cells.

Bacterial cells (∼3.5 × 10^5^ CFU) from a mid-log culture were treated with 100 μg/ml of purified RAD2-DpK2. Human sera (Sigma-Aldrich catalog no. S7023) were prewarmed to either 37°C (active) or 50°C (heat inactivated) for 30 min and then added to enzyme-treated cells (80% final concentration) and incubated at 37°C for 3 h. Viable bacterial counts were determined before and after incubation with serum.

### Data availability.

The RAD2 genome sequence data were deposited in GenBank (https://www.ncbi.nlm.nih.gov/genbank/) under accession number MW655991. RAD2 phage challenge TraDIS sequence data were deposited in the European Nucleotide Archive (https://www.ebi.ac.uk/ena/browser/home) under BioProject study accession number PRJEB25275. The DpK2 cryo-EM density map and PDB coordinates were deposited in the Electron Microscopy Data Bank (https://www.ebi.ac.uk/pdbe/emdb/) and the PDB (https://www.rcsb.org/) under accession codes EMD-23608 and 7LZJ, respectively.

## References

[1] Podschun R, Ullmann U. 1998. *Klebsiella* spp. as nosocomial pathogens: epidemiology, taxonomy, typing methods, and pathogenicity factors. Clin Microbiol Rev 11:589–603. doi:10.1128/CMR.11.4.589. 9767057 PMC88898

[2] Holt KE, Wertheim H, Zadoks RN, Baker S, Whitehouse CA, Dance D, Jenney A, Connor TR, Hsu LY, Severin J, Brisse S, Cao H, Wilksch J, Gorrie C, Schultz MB, Edwards DJ, Nguyen KV, Nguyen TV, Dao TT, Mensink M, Minh VL, Nhu NT, Schultsz C, Kuntaman K, Newton PN, Moore CE, Strugnell RA, Thomson NR. 2015. Genomic analysis of diversity, population structure, virulence, and antimicrobial resistance in *Klebsiella pneumoniae*, an urgent threat to public health. Proc Natl Acad Sci USA 112:E3574–E3581. doi:10.1073/pnas.1501049112. 26100894 PMC4500264

[3] Paczosa MK, Mecsas J. 2016. *Klebsiella pneumoniae*: going on the offense with a strong defense. Microbiol Mol Biol Rev 80:629–661. doi:10.1128/MMBR.00078-15. 27307579 PMC4981674

[4] Sahly H, Podschun R, Ullmann U. 2000. *Klebsiella* infections in the immunocompromised host. Adv Exp Med Biol 479:237–249. doi:10.1007/0-306-46831-X_21. 10897425

[5] Gomez-Simmonds A, Uhlemann AC. 2017. Clinical implications of genomic adaptation and evolution of carbapenem-resistant *Klebsiella pneumoniae*. J Infect Dis 215:S18–S27. doi:10.1093/infdis/jiw378. 28375514 PMC5853309

[6] Pomakova DK, Hsiao CB, Beanan JM, Olson R, MacDonald U, Keynan Y, Russo TA. 2012. Clinical and phenotypic differences between classic and hypervirulent *Klebsiella pneumonia*: an emerging and under-recognized pathogenic variant. Eur J Clin Microbiol Infect Dis 31:981–989. doi:10.1007/s10096-011-1396-6. 21918907

[7] Wang JH, Liu YC, Lee SS, Yen MY, Chen YS, Wang JH, Wann SR, Lin HH. 1998. Primary liver abscess due to *Klebsiella pneumoniae* in Taiwan. Clin Infect Dis 26:1434–1438. doi:10.1086/516369. 9636876

[8] Tacconelli E, Carrara E, Savoldi A, Harbarth S, Mendelson M, Monnet DL, Pulcini C, Kahlmeter G, Kluytmans J, Carmeli Y, Ouellette M, Outterson K, Patel J, Cavaleri M, Cox EM, Houchens CR, Grayson ML, Hansen P, Singh N, Theuretzbacher U, Magrini N, WHO Pathogens Priority List Working Group. 2018. Discovery, research, and development of new antibiotics: the WHO priority list of antibiotic-resistant bacteria and tuberculosis. Lancet Infect Dis 18:318–327. doi:10.1016/S1473-3099(17)30753-3. 29276051

[9] Nobrega FL, Vlot M, de Jonge PA, Dreesens LL, Beaumont HJE, Lavigne R, Dutilh BE, Brouns SJJ. 2018. Targeting mechanisms of tailed bacteriophages. Nat Rev Microbiol 16:760–773. doi:10.1038/s41579-018-0070-8. 30104690

[10] Pan YJ, Lin TL, Chen CC, Tsai YT, Cheng YH, Chen YY, Hsieh PF, Lin YT, Wang JT. 2017. *Klebsiella* phage phiK64-1 encodes multiple depolymerases for multiple host capsular types. J Virol 91:e02457-16. doi:10.1128/JVI.02457-16. 28077636 PMC5331798

[11] Plattner M, Shneider MM, Arbatsky NP, Shashkov AS, Chizhov AO, Nazarov S, Prokhorov NS, Taylor NMI, Buth SA, Gambino M, Gencay YE, Brondsted L, Kutter EM, Knirel YA, Leiman PG. 2019. Structure and function of the branched receptor-binding complex of bacteriophage CBA120. J Mol Biol 431:3718–3739. doi:10.1016/j.jmb.2019.07.022. 31325442

[12] Squeglia F, Maciejewska B, Łątka A, Ruggiero A, Briers Y, Drulis-Kawa Z, Berisio R. 2020. Structural and functional studies of a *Klebsiella* phage capsule depolymerase tailspike: mechanistic insights into capsular degradation. Structure 28:613–624.e4. doi:10.1016/j.str.2020.04.015. 32386574

[13] Pan YJ, Lin TL, Chen CT, Chen YY, Hsieh PF, Hsu CR, Wu MC, Wang JT. 2015. Genetic analysis of capsular polysaccharide synthesis gene clusters in 79 capsular types of *Klebsiella* spp. Sci Rep 5:15573. doi:10.1038/srep15573. 26493302 PMC4616057

[14] Wyres KL, Wick RR, Gorrie C, Jenney A, Follador R, Thomson NR, Holt KE. 2016. Identification of *Klebsiella* capsule synthesis loci from whole genome data. Microb Genom 2:e000102. doi:10.1099/mgen.0.000102. 28348840 PMC5359410

[15] Shon AS, Bajwa RP, Russo TA. 2013. Hypervirulent (hypermucoviscous) *Klebsiella pneumoniae*: a new and dangerous breed. Virulence 4:107–118. doi:10.4161/viru.22718. 23302790 PMC3654609

[16] Latka A, Maciejewska B, Majkowska-Skrobek G, Briers Y, Drulis-Kawa Z. 2017. Bacteriophage-encoded virion-associated enzymes to overcome the carbohydrate barriers during the infection process. Appl Microbiol Biotechnol 101:3103–3119. doi:10.1007/s00253-017-8224-6. 28337580 PMC5380687

[17] Pires DP, Oliveira H, Melo LD, Sillankorva S, Azeredo J. 2016. Bacteriophage-encoded depolymerases: their diversity and biotechnological applications. Appl Microbiol Biotechnol 100:2141–2151. doi:10.1007/s00253-015-7247-0. 26767986

[18] Latka A, Leiman PG, Drulis-Kawa Z, Briers Y. 2019. Modeling the architecture of depolymerase-containing receptor binding proteins in *Klebsiella* phages. Front Microbiol 10:2649. doi:10.3389/fmicb.2019.02649. 31803168 PMC6872550

[19] Leiman PG, Molineux IJ. 2008. Evolution of a new enzyme activity from the same motif fold. Mol Microbiol 69:287–290. doi:10.1111/j.1365-2958.2008.06241.x. 18433454 PMC2574927

[20] Thung TY, White ME, Dai W, Wilksch JJ, Bamert RS, Rocker A, Stubenrauch CJ, Williams D, Huang C, Schittelhelm R, Barr JJ, Jameson E, McGowan S, Zhang Y, Wang J, Dunstan RA, Lithgow T. 2021. The component parts of bacteriophage virions accurately defined by a machine-learning approach built on evolutionary features. bioRxiv. doi:10.1101/2021.02.28.433281. PMC8269216 34042467

[21] Lin TL, Hsieh PF, Huang YT, Lee WC, Tsai YT, Su PA, Pan YJ, Hsu CR, Wu MC, Wang JT. 2014. Isolation of a bacteriophage and its depolymerase specific for K1 capsule of *Klebsiella pneumoniae*: implication in typing and treatment. J Infect Dis 210:1734–1744. doi:10.1093/infdis/jiu332. 25001459

[22] Majkowska-Skrobek G, Łątka A, Berisio R, Maciejewska B, Squeglia F, Romano M, Lavigne R, Struve C, Drulis-Kawa Z. 2016. Capsule-targeting depolymerase, derived from *Klebsiella* KP36 phage, as a tool for the development of anti-virulent strategy. Viruses 8. doi:10.3390/v8120324. PMC5192385 27916936

[23] Majkowska-Skrobek G, Latka A, Berisio R, Squeglia F, Maciejewska B, Briers Y, Drulis-Kawa Z. 2018. Phage-borne depolymerases decrease *Klebsiella pneumoniae* resistance to innate defense mechanisms. Front Microbiol 9:2517. doi:10.3389/fmicb.2018.02517. 30405575 PMC6205948

[24] V Volozhantsev N, M Shpirt A, I Borzilov A, V Komisarova E, M Krasilnikova V, S Shashkov A, V Verevkin V, A Knirel Y. 2020. Characterization and therapeutic potential of bacteriophage-encoded polysaccharide depolymerases with beta galactosidase activity against *Klebsiella pneumoniae* K57 capsular type. Antibiotics (Basel) 9:732. doi:10.3390/antibiotics9110732. 33113762 PMC7693772

[25] Chan BK, Abedon ST. 2015. Bacteriophages and their enzymes in biofilm control. Curr Pharm Des 21:85–99. doi:10.2174/1381612820666140905112311. 25189866

[26] Topka-Bielecka G, Dydecka A, Necel A, Bloch S, Nejman-Faleńczyk B, Węgrzyn G, Węgrzyn A. 2021. Bacteriophage-derived depolymerases against bacterial biofilm. Antibiotics (Basel) 10:175. doi:10.3390/antibiotics10020175. 33578658 PMC7916357

[27] Chan PP, Lowe TM. 2019. tRNAscan-SE: searching for tRNA genes in genomic sequences. Methods Mol Biol 1962:1–14. doi:10.1007/978-1-4939-9173-0_1. 31020551 PMC6768409

[28] Garneau JR, Depardieu F, Fortier LC, Bikard D, Monot M. 2017. PhageTerm: a tool for fast and accurate determination of phage termini and packaging mechanism using next-generation sequencing data. Sci Rep 7:8292. doi:10.1038/s41598-017-07910-5. 28811656 PMC5557969

[29] Cai R, Wang Z, Wang G, Zhang H, Cheng M, Guo Z, Ji Y, Xi H, Wang X, Xue Y, Ur Rahman S, Sun C, Feng X, Lei L, Tong Y, Han W, Gu J. 2019. Biological properties and genomics analysis of vB_KpnS_GH-K3, a *Klebsiella* phage with a putative depolymerase-like protein. Virus Genes 55:696–706. doi:10.1007/s11262-019-01681-z. 31254238

[30] Dorman MJ, Feltwell T, Goulding DA, Parkhill J, Short FL. 2018. The capsule regulatory network of *Klebsiella pneumoniae* defined by density-TraDISort. mBio 9:e01863-18. doi:10.1128/mBio.01863-18. 30459193 PMC6247091

[31] Arakawa Y, Wacharotayankun R, Nagatsuka T, Ito H, Kato N, Ohta M. 1995. Genomic organization of the *Klebsiella pneumoniae* cps region responsible for serotype K2 capsular polysaccharide synthesis in the virulent strain Chedid. J Bacteriol 177:1788–1796. doi:10.1128/jb.177.7.1788-1796.1995. 7896702 PMC176807

[32] Bailey MJ, Hughes C, Koronakis V. 1997. RfaH and the ops element, components of a novel system controlling bacterial transcription elongation. Mol Microbiol 26:845–851. doi:10.1046/j.1365-2958.1997.6432014.x. 9426123

[33] Bachman MA, Breen P, Deornellas V, Mu Q, Zhao L, Wu W, Cavalcoli JD, Mobley HL. 2015. Genome-wide identification of *Klebsiella pneumoniae* fitness genes during lung infection. mBio 6:e00775-15. doi:10.1128/mBio.00775-15. 26060277 PMC4462621

[34] Sutherland IW. 1985. Biosynthesis and composition of gram-negative bacterial extracellular and wall polysaccharides. Annu Rev Microbiol 39:243–270. doi:10.1146/annurev.mi.39.100185.001331. 3904602

[35] Chang HY, Lee JH, Deng WL, Fu TF, Peng HL. 1996. Virulence and outer membrane properties of a galU mutant of *Klebsiella pneumoniae* CG43. Microb Pathog 20:255–261. doi:10.1006/mpat.1996.0024. 8861391

[36] Mike LA, Stark AJ, Forsyth VS, Vornhagen J, Smith SN, Bachman MA, Mobley HLT. 2021. A systematic analysis of hypermucoviscosity and capsule reveals distinct and overlapping genes that impact *Klebsiella pneumoniae* fitness. PLoS Pathog 17:e1009376. doi:10.1371/journal.ppat.1009376. 33720976 PMC7993769

[37] Clements A, Gaboriaud F, Duval JF, Farn JL, Jenney AW, Lithgow T, Wijburg OL, Hartland EL, Strugnell RA. 2008. The major surface-associated saccharides of *Klebsiella pneumoniae* contribute to host cell association. PLoS One 3:e3817. doi:10.1371/journal.pone.0003817. 19043570 PMC2583945

[38] Frirdich E, Vinogradov E, Whitfield C. 2004. Biosynthesis of a novel 3-deoxy-d-manno-oct-2-ulosonic acid-containing outer core oligosaccharide in the lipopolysaccharide of *Klebsiella pneumoniae*. J Biol Chem 279:27928–27940. doi:10.1074/jbc.M402549200. 15090547

[39] Regue M, Izquierdo L, Fresno S, Jimenez N, Pique N, Corsaro MM, Parrilli M, Naldi T, Merino S, Tomas JM. 2005. The incorporation of glucosamine into enterobacterial core lipopolysaccharide: two enzymatic steps are required. J Biol Chem 280:36648–36656. doi:10.1074/jbc.M506278200. 16131489

[40] Fresno S, Jimenez N, Canals R, Merino S, Corsaro MM, Lanzetta R, Parrilli M, Pieretti G, Regue M, Tomas JM. 2007. A second galacturonic acid transferase is required for core lipopolysaccharide biosynthesis and complete capsule association with the cell surface in *Klebsiella pneumoniae*. J Bacteriol 189:1128–1137. doi:10.1128/JB.01489-06. 17142396 PMC1797326

[41] Vinogradov E, Cedzynski M, Ziolkowski A, Swierzko A. 2001. The structure of the core region of the lipopolysaccharide from *Klebsiella pneumoniae* O3. 3-deoxy-alpha-d-manno-octulosonic acid (alpha-Kdo) residue in the outer part of the core, a common structural element of *Klebsiella pneumoniae* O1, O2, O3, O4, O5, O8, and O12 lipopolysaccharides. Eur J Biochem 268:1722–1729. doi:10.1046/j.1432-1033.2001.02047.x. 11248692

[42] Vinogradov E, Perry MB. 2001. Structural analysis of the core region of the lipopolysaccharides from eight serotypes of *Klebsiella pneumoniae*. Carbohydr Res 335:291–296. doi:10.1016/s0008-6215(01)00216-6. 11595223

[43] Steinbacher S, Baxa U, Miller S, Weintraub A, Seckler R, Huber R. 1996. Crystal structure of phage P22 tailspike protein complexed with *Salmonella* sp. O-antigen receptors. Proc Natl Acad Sci USA 93:10584–10588. doi:10.1073/pnas.93.20.10584. 8855221 PMC38196

[44] Holm L, Kaariainen S, Wilton C, Plewczynski D. 2006. Using Dali for structural comparison of proteins. Curr Protoc Bioinformatics Chapter 5:Unit 5.5. doi:10.1002/0471250953.bi0505s14. 18428766

[45] Holm L. 2020. DALI and the persistence of protein shape. Protein Sci 29:128–140. doi:10.1002/pro.3749. 31606894 PMC6933842

[46] Westphal OJK. 1965. Bacterial lipopolysaccharides extraction with phenol-water and further applications of the procedure. Methods Carbohydr Chem 5:83–91.

[47] Al-Hakim A, Linhardt RJ. 1990. Isolation and recovery of acidic oligosaccharides from polyacrylamide gels by semi-dry electrotransfer. Electrophoresis 11:23–28. doi:10.1002/elps.1150110106. 1690641

[48] Mellitzer A, Glieder A, Weis R, Reisinger C, Flicker K. 2012. Sensitive high-throughput screening for the detection of reducing sugars. Biotechnol J 7:155–162. doi:10.1002/biot.201100001. 21538898

[49] Kassa T, Chhibber S. 2012. Thermal treatment of the bacteriophage lysate of *Klebsiella pneumoniae* B5055 as a step for the purification of capsular depolymerase enzyme. J Virol Methods 179:135–141. doi:10.1016/j.jviromet.2011.10.011. 22036659

[50] Yan J, Mao J, Mao J, Xie J. 2014. Bacteriophage polysaccharide depolymerases and biomedical applications. BioDrugs 28:265–274. doi:10.1007/s40259-013-0081-y. 24352884

[51] Davies G, Henrissat B. 1995. Structures and mechanisms of glycosyl hydrolases. Structure 3:853–859. doi:10.1016/S0969-2126(01)00220-9. 8535779

[52] Corsaro MM, De Castro C, Naldi T, Parrilli M, Tomas JM, Regue M. 2005. 1H and 13C NMR characterization and secondary structure of the K2 polysaccharide of *Klebsiella pneumoniae* strain 52145. Carbohydr Res 340:2212–2217. doi:10.1016/j.carres.2005.07.006. 16054607

[53] Maneval WE. 1941. Staining bacteria and yeasts with acid dyes. Stain Technol 16:13–19. doi:10.3109/10520294109106189.

[54] Wang H, Wilksch JJ, Lithgow T, Strugnell RA, Gee ML. 2013. Nanomechanics measurements of live bacteria reveal a mechanism for bacterial cell protection: the polysaccharide capsule in *Klebsiella* is a responsive polymer hydrogel that adapts to osmotic stress. Soft Matter 9:7560–7567. doi:10.1039/c3sm51325d.

[55] Hardy JM, Dunstan RA, Grinter R, Belousoff MJ, Wang J, Pickard D, Venugopal H, Dougan G, Lithgow T, Coulibaly F. 2020. The architecture and stabilisation of flagellotropic tailed bacteriophages. Nat Commun 11:3748. doi:10.1038/s41467-020-17505-w. 32719311 PMC7385642

[56] Heller K, Braun V. 1982. Polymannose O-antigens of *Escherichia coli*, the binding sites for the reversible adsorption of bacteriophage T5+ via the L-shaped tail fibers. J Virol 41:222–227. doi:10.1128/JVI.41.1.222-227.1982. 7045389 PMC256742

[57] Hendrix RW, Duda RL. 1992. Bacteriophage lambda PaPa: not the mother of all lambda phages. Science 258:1145–1148. doi:10.1126/science.1439823. 1439823

[58] Hug H, Hausmann R, Liebeschuetz J, Ritchie DA. 1986. In vitro packaging of foreign DNA into heads of bacteriophage T1. J Gen Virol 67:333–343. doi:10.1099/0022-1317-67-2-333. 3003241

[59] Schade SZ, Adler J, Ris H. 1967. How bacteriophage chi attacks motile bacteria. J Virol 1:599–609. doi:10.1128/JVI.1.3.599-609.1967. 4918241 PMC375288

[60] Fresno S, Jimenez N, Izquierdo L, Merino S, Corsaro MM, De Castro C, Parrilli M, Naldi T, Regue M, Tomas JM. 2006. The ionic interaction of *Klebsiella pneumoniae* K2 capsule and core lipopolysaccharide. Microbiology (Reading) 152:1807–1818. doi:10.1099/mic.0.28611-0. 16735743

[61] Opoku-Temeng C, Kobayashi SD, DeLeo FR. 2019. *Klebsiella pneumoniae* capsule polysaccharide as a target for therapeutics and vaccines. Comput Struct Biotechnol J 17:1360–1366. doi:10.1016/j.csbj.2019.09.011. 31762959 PMC6861629

[62] Short FL, Di Sario G, Reichmann NT, Kleanthous C, Parkhill J, Taylor PW. 2020. Genomic profiling reveals distinct routes to complement resistance in *Klebsiella pneumoniae*. Infect Immun 88:e00043-20. doi:10.1128/IAI.00043-20. 32513855 PMC7375759

[63] Hsieh PF, Lin HH, Lin TL, Chen YY, Wang JT. 2017. Two T7-like bacteriophages, K5-2 and K5-4, each encodes two capsule depolymerases: isolation and functional characterization. Sci Rep 7:4624. doi:10.1038/s41598-017-04644-2. 28676686 PMC5496888

[64] Dunstan RA, Pickard D, Dougan S, Goulding D, Cormie C, Hardy J, Li F, Grinter R, Harcourt K, Yu L, Song J, Schreiber F, Choudhary J, Clare S, Coulibaly F, Strugnell RA, Dougan G, Lithgow T. 2019. The flagellotropic bacteriophage YSD1 targets Salmonella Typhi with a Chi-like protein tail fibre. Mol Microbiol 112:1831–1846. doi:10.1111/mmi.14396. 31556164

[65] Hunt M, Gall A, Ong SH, Brener J, Ferns B, Goulder P, Nastouli E, Keane JA, Kellam P, Otto TD. 2015. IVA: accurate de novo assembly of RNA virus genomes. Bioinformatics 31:2374–2376. doi:10.1093/bioinformatics/btv120. 25725497 PMC4495290

[66] Seemann T. 2014. Prokka: rapid prokaryotic genome annotation. Bioinformatics 30:2068–2069. doi:10.1093/bioinformatics/btu153. 24642063

[67] Altschul SF, Gish W, Miller W, Myers EW, Lipman DJ. 1990. Basic local alignment search tool. J Mol Biol 215:403–410. doi:10.1016/S0022-2836(05)80360-2. 2231712

[68] Zimmermann L, Stephens A, Nam SZ, Rau D, Kubler J, Lozajic M, Gabler F, Soding J, Lupas AN, Alva V. 2018. A completely reimplemented MPI bioinformatics toolkit with a new HHpred server at its core. J Mol Biol 430:2237–2243. doi:10.1016/j.jmb.2017.12.007. 29258817

[69] Carver T, Thomson N, Bleasby A, Berriman M, Parkhill J. 2009. DNAPlotter: circular and linear interactive genome visualization. Bioinformatics 25:119–120. doi:10.1093/bioinformatics/btn578. 18990721 PMC2612626

[70] Nishimura Y, Yoshida T, Kuronishi M, Uehara H, Ogata H, Goto S. 2017. ViPTree: the viral proteomic tree server. Bioinformatics 33:2379–2380. doi:10.1093/bioinformatics/btx157. 28379287

[71] Barquist L, Mayho M, Cummins C, Cain AK, Boinett CJ, Page AJ, Langridge GC, Quail MA, Keane JA, Parkhill J. 2016. The TraDIS toolkit: sequencing and analysis for dense transposon mutant libraries. Bioinformatics 32:1109–1111. doi:10.1093/bioinformatics/btw022. 26794317 PMC4896371

[72] Goh KGK, Phan MD, Forde BM, Chong TM, Yin WF, Chan KG, Ulett GC, Sweet MJ, Beatson SA, Schembri MA. 2017. Genome-wide discovery of genes required for capsule production by uropathogenic *Escherichia coli*. mBio 8:e01558-17. doi:10.1128/mBio.01558-17. 29066548 PMC5654933

[73] Wright A, Deane-Alder K, Marschall E, Bamert R, Venugopal H, Lithgow T, Lupton DW, Belousoff MJ. 2020. Characterization of the core ribosomal binding region for the oxazolidone family of antibiotics using cryo-EM. ACS Pharmacol Transl Sci 3:425–432. doi:10.1021/acsptsci.0c00041. 32566908 PMC7296538

[74] Zivanov J, Nakane T, Forsberg BO, Kimanius D, Hagen WJ, Lindahl E, Scheres SH. 2018. New tools for automated high-resolution cryo-EM structure determination in RELION-3. Elife 7. doi:10.7554/eLife.42166. PMC6250425 30412051

[75] Fernandez-Leiro R, Scheres SHW. 2017. A pipeline approach to single-particle processing in RELION. Acta Crystallogr D Struct Biol 73:496–502. doi:10.1107/S2059798316019276. 28580911 PMC5458491

[76] Scheres SH. 2016. Processing of Structurally Heterogeneous Cryo-EM Data in RELION. Methods Enzymol 579:125–157. doi:10.1016/bs.mie.2016.04.012. 27572726

[77] Kimanius D, Forsberg BO, Scheres SHW, Lindahl E. 2016. Accelerated cryo-EM structure determination with parallelisation using GPUs in RELION-2. Elife 5:e18722. doi:10.7554/eLife.18722. 27845625 PMC5310839

[78] Scheres SH. 2015. Semi-automated selection of cryo-EM particles in RELION-1.3. J Struct Biol 189:114–122. doi:10.1016/j.jsb.2014.11.010. 25486611 PMC4318617

[79] Scheres SH. 2012. RELION: implementation of a Bayesian approach to cryo-EM structure determination. J Struct Biol 180:519–530. doi:10.1016/j.jsb.2012.09.006. 23000701 PMC3690530

[80] Punjani A, Rubinstein JL, Fleet DJ, Brubaker MA. 2017. cryoSPARC: algorithms for rapid unsupervised cryo-EM structure determination. Nat Methods 14:290–296. doi:10.1038/nmeth.4169. 28165473

[81] Cowtan K. 2006. The Buccaneer software for automated model building. 1. Tracing protein chains. Acta Crystallogr D Biol Crystallogr 62:1002–1011. doi:10.1107/S0907444906022116. 16929101

[82] Emsley P, Lohkamp B, Scott WG, Cowtan K. 2010. Features and development of Coot. Acta Crystallogr D Biol Crystallogr 66:486–501. doi:10.1107/S0907444910007493. 20383002 PMC2852313

[83] Afonine PV, Poon BK, Read RJ, Sobolev OV, Terwilliger TC, Urzhumtsev A, Adams PD. 2018. Real-space refinement in PHENIX for cryo-EM and crystallography. Acta Crystallogr D Struct Biol 74:531–544. doi:10.1107/S2059798318006551. 29872004 PMC6096492

[84] Adams PD, Afonine PV, Bunkoczi G, Chen VB, Davis IW, Echols N, Headd JJ, Hung LW, Kapral GJ, Grosse-Kunstleve RW, McCoy AJ, Moriarty NW, Oeffner R, Read RJ, Richardson DC, Richardson JS, Terwilliger TC, Zwart PH. 2010. PHENIX: a comprehensive Python-based system for macromolecular structure solution. Acta Crystallogr D Biol Crystallogr 66:213–221. doi:10.1107/S0907444909052925. 20124702 PMC2815670

[85] Creek DJ, Jankevics A, Burgess KE, Breitling R, Barrett MP. 2012. IDEOM: an Excel interface for analysis of LC-MS-based metabolomics data. Bioinformatics 28:1048–1049. doi:10.1093/bioinformatics/bts069. 22308147

[86] Holman JD, Tabb DL, Mallick P. 2014. Employing ProteoWizard to convert raw mass spectrometry data. Curr Protoc Bioinformatics 46:13.24.1–9. doi:10.1002/0471250953.bi1324s46. PMC4113728 24939128

[87] Smith CA, Want EJ, O'Maille G, Abagyan R, Siuzdak G. 2006. XCMS: processing mass spectrometry data for metabolite profiling using nonlinear peak alignment, matching, and identification. Anal Chem 78:779–787. doi:10.1021/ac051437y. 16448051

